# 1-(4-Methyl­benz­yl)-1*H*-benzimidazol-2(3*H*)-one

**DOI:** 10.1107/S1600536812050726

**Published:** 2012-12-22

**Authors:** Dounia Belaziz, Youssef Kandri Rodi, Fouad Ouazzani Chahdi, El Mokhtar Essassi, Mohamed Saadi, Lahcen El Ammari

**Affiliations:** a Laboratoire de Chimie Organique Appliquée, Université Sidi Mohamed, Ben Abdallah, Faculté des Sciences et Techniques, Route d’immouzzer, BP 2202 Fès, Morocco; b Laboratoire de Chimie Organique Hétérocyclique URAC21, Faculté des Sciences, Université Mohammed V-Agdal, Avenue Ibn Battouta, BP 1014, Rabat, Morocco; c Institute of Nanmaterials and Nanotechnology, MASCIR, Rabat, Morocco; d Laboratoire de Chimie du Solide Appliquée, Faculté des Sciences, Université Mohammed V-Agdal, Avenue Ibn Battouta, BP 1014, Rabat, Morocco

## Abstract

In the title compound, C_15_H_14_N_2_O, the fused five- and six-membered ring system is essentially planar, the maximum deviation from the mean plane being 0.009 (1) Å. The benzimidazol-2(3*H*)-one residue is nearly perpendicular to the benzyl ring, forming a dihedral angle of 77.41 (6)°. In the crystal, inversion dimers are formed by pairs of N—H⋯O hydrogen bonds; these dimers are linked by weak C—H⋯O inter­actions into a two-dimensional array in the (102) plane.

## Related literature

For pharmacological and biochemical properties of benzimidazole derivatives, see: Lee *et al.* (2004 ▶); Deligeorgiev *et al.* (2011 ▶); Scott *et al.* (2002 ▶); Gothelf *et al.* (1998 ▶). For related structures, see: Belaziz *et al.* (2012 ▶); Ouzidan *et al.* (2011 ▶).
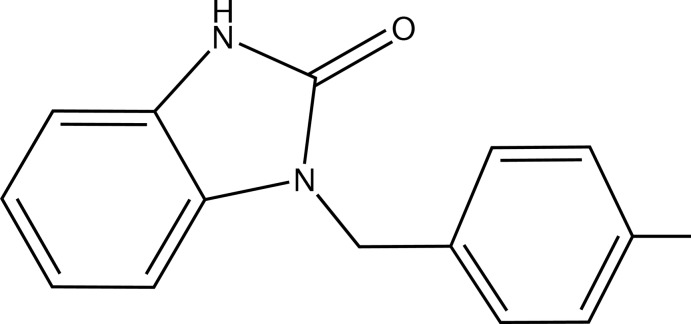


## Experimental

### 

#### Crystal data

C_15_H_14_N_2_O
*M* *_r_* = 238.28
Monoclinic, 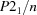

*a* = 12.5585 (5) Å
*b* = 5.7181 (2) Å
*c* = 17.4153 (7) Å
β = 95.277 (2)°
*V* = 1245.31 (8) Å^3^
*Z* = 4
Mo *K*α radiation
μ = 0.08 mm^−1^
*T* = 296 K
0.51 × 0.42 × 0.15 mm

#### Data collection

Bruker X8 APEXII diffractometer
16486 measured reflections
3211 independent reflections
2157 reflections with *I* > 2σ(*I*)
*R*_int_ = 0.029

#### Refinement

*R*[*F* ^2^ > 2σ(*F* ^2^)] = 0.044
*wR*(*F* ^2^) = 0.122
*S* = 1.02
3211 reflections
164 parameters
H-atom parameters constrained
Δρ_max_ = 0.17 e Å^−3^
Δρ_min_ = −0.15 e Å^−3^

### 

Data collection: *APEX2* (Bruker, 2009 ▶); cell refinement: *SAINT* (Bruker, 2009 ▶); data reduction: *SAINT*; program(s) used to solve structure: *SHELXS97* (Sheldrick, 2008 ▶); program(s) used to refine structure: *SHELXL97* (Sheldrick, 2008 ▶); molecular graphics: *ORTEP-3 for Windows* (Farrugia, 2012 ▶); software used to prepare material for publication: *PLATON* (Spek, 2009 ▶) and *publCIF* (Westrip,2010 ▶).

## Supplementary Material

Crystal structure: contains datablock(s) I, global. DOI: 10.1107/S1600536812050726/tk5182sup1.cif

Structure factors: contains datablock(s) I. DOI: 10.1107/S1600536812050726/tk5182Isup2.hkl

Supplementary material file. DOI: 10.1107/S1600536812050726/tk5182Isup3.cml

Additional supplementary materials:  crystallographic information; 3D view; checkCIF report

## Figures and Tables

**Table 1:** Hydrogen-bond geometry (Å, °)

*D*—H⋯*A*	*D*—H	H⋯*A*	*D*⋯*A*	*D*—H⋯*A*
N1—H1*N*1⋯O1^i^	0.94	1.91	2.8317 (15)	166
C15—H15*C*⋯O1^ii^	0.96	2.58	3.514 (2)	165
C8—H8*A*⋯O1^iii^	0.97	2.61	3.5504 (18)	164

Symmetry codes: (i) 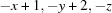; (ii) 
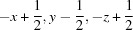
; (iii) 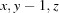.

**Fig. 1.**
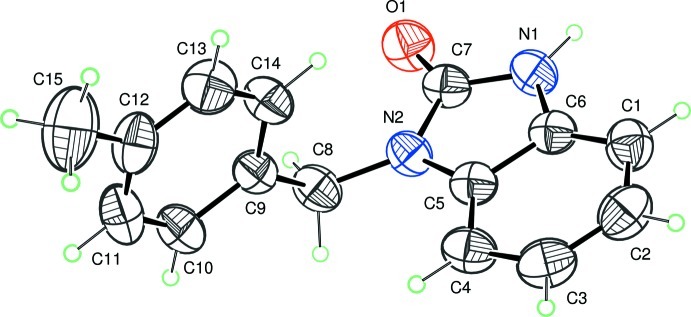
Molecular structure of the title compound with the atom-labelling scheme. Displacement ellipsoids are drawn at the 50% probability level. H atoms are represented as small circles.

**Fig. 2.**
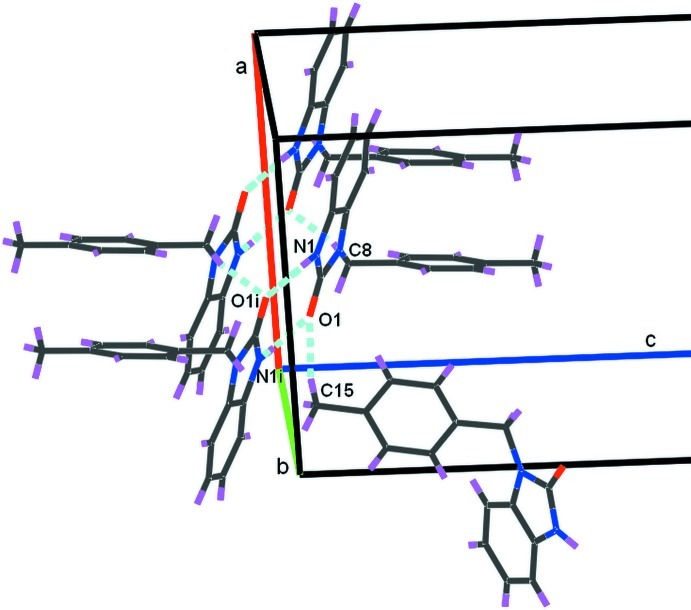
Portion of the unit cell showing intermolecular interactions (dashed lines) as detailed in Table 1.

## supplementary crystallographic information

### Comment

The development of benzimidazole derivatives has experienced in recent years, a considerable expansion following reports of biological activities presented by this type of compound. Benzimidazole derivatives are endowed with anti-viral, anti-ulcer, anti-hypertensive and anti-cancer activities (Lee *et al.*, 2004; Deligeorgiev *et al.*, 2011; Scott *et al.*, 2002). Heterocycles containing the benzimidazole nucleus are also antagonists of a number of biological receptors, namely angiotensin II and prostaglandin D2 (Gothelf *et al.*, 1998).

In a previous study, we reacted benzimidazol-2-one with dodecyl bromide in the presence of a catalytic quantity of tetra-*n*-butylammonium bromide under mild conditions to form 1-dodecyl-1*H*-benzimidazol-2(3*H*)-one (Belaziz *et al.*, 2012; Ouzidan *et al.*, 2011). The study is extended to the synthesis of new benzimidazol-2-one derivative by action of methylbenzyl bromide with 1*H*-benzimidazol-2(3*H*)-one to form the title compound (Scheme 1).

The crystal structure of the title compound, C_15_H_14_N_2_O, is built up from two fused five- and six-membered rings (C1-C7,N1,N2,O1) linked to (C8-C15) the *p*-methyl-benzyl residue as shown in Fig. 1. The fused-ring system is essentially planar, with the maximum deviation of 0.009 (1) Å for the N2 atom. The dihedral angle between the benzimidazol-2(3*H*-one system and the (C9 to C14) benzyl ring is 77.41 (6)°.

In the crystal, inversion dimers are linked by N1—H1N···O1 hydrogen bonds. These are linked by weak C8–H8A···O1 and C15–H15C···O1 non-classic hydrogen bonds to form a layer parallel to (1 0 2); see Fig. 2 and Table 1.

### Experimental

To 1*H*-benzimidazol-2(3*H*)-one (0.2 g, 1.49 mmol), potassium carbonate (0.41 g, 3 mmol) and tetra-*n*-butylammonium bromide (0.05 g, 0.15 mmol) in DMF (15 ml) was added methyl benzyl bromide (0.33 g, 1.78 mmol). Stirring was continued at room temperature for 6 h. The salt was removed by filtration and the filtrate concentrated under reduced pressure. The residue was separated by chromatography on a column of silica gel with ethyl acetate/hexane (1/2) as eluent. The compound was recrystallized from hexane to give colorless crystals.

### Refinement

H atoms were located in a difference map and treated as riding with N—H = 0.94 Å, C—H = 0.93 Å (aromatic), C—H = 0.97 Å (methylene) and C—H = 0.96 Å (methyl), and with *U*_iso_(H) = 1.2 *U*_eq_ (N, aromatic-C, methylene-C) and *U*_iso_(H) = 1.5 *U*_eq_(methyl-C). Two reflections, *i.e*. (-1 0 1) and (1 0 1), were omitted owing to poor agreement.

### Crystal data

**Table:**

C_15_H_14_N_2_O	*F*(000) = 504
*M**_r_* = 238.28	*D*_x_ = 1.271 Mg m^−^^3^
Monoclinic, *P*2_1_/*n*	Melting point: 456 K
Hall symbol: -P 2yn	Mo *K*α radiation, λ = 0.71073 Å
*a* = 12.5585 (5) Å	Cell parameters from 3211 reflections
*b* = 5.7181 (2) Å	θ = 3.3–28.7°
*c* = 17.4153 (7) Å	µ = 0.08 mm^−^^1^
β = 95.277 (2)°	*T* = 296 K
*V* = 1245.31 (8) Å^3^	Block, colourless
*Z* = 4	0.51 × 0.42 × 0.15 mm

### Data collection

**Table:**

Bruker X8 APEXII diffractometer	2157 reflections with *I* > 2σ(*I*)
Radiation source: fine-focus sealed tube	*R*_int_ = 0.029
Graphite monochromator	θ_max_ = 28.7°, θ_min_ = 3.3°
φ and ω scans	*h* = −16→10
16486 measured reflections	*k* = −7→7
3211 independent reflections	*l* = −23→23

### Refinement

**Table:**

Refinement on *F*^2^	Secondary atom site location: difference Fourier map
Least-squares matrix: full	Hydrogen site location: inferred from neighbouring sites
*R*[*F*^2^ > 2σ(*F*^2^)] = 0.044	H-atom parameters constrained
*wR*(*F*^2^) = 0.122	*w* = 1/[σ^2^(*F*_o_^2^) + (0.0511*P*)^2^ + 0.2271*P*] where *P* = (*F*_o_^2^ + 2*F*_c_^2^)/3
*S* = 1.02	(Δ/σ)_max_ < 0.001
3211 reflections	Δρ_max_ = 0.17 e Å^−^^3^
164 parameters	Δρ_min_ = −0.15 e Å^−^^3^
0 restraints	Extinction correction: *SHELXL97* (Sheldrick, 2008), Fc^*^=kFc[1+0.001xFc^2^λ^3^/sin(2θ)]^-1/4^
Primary atom site location: structure-invariant direct methods	Extinction coefficient: 0.0047 (19)

### Special details

**Table:**

Geometry. All e.s.d.'s (except the e.s.d. in the dihedral angle between two l.s. planes) are estimated using the full covariance matrix. The cell e.s.d.'s are taken into account individually in the estimation of e.s.d.'s in distances, angles and torsion angles; correlations between e.s.d.'s in cell parameters are only used when they are defined by crystal symmetry. An approximate (isotropic) treatment of cell e.s.d.'s is used for estimating e.s.d.'s involving l.s. planes.
Refinement. Refinement of *F*^2^ against all reflections. The weighted *R*-factor *wR* and goodness of fit *S* are based on *F*^2^, conventional *R*-factors *R* are based on *F*, with *F* set to zero for negative *F*^2^. The threshold expression of *F*^2^ > σ(*F*^2^) is used only for calculating *R*-factors(gt) *etc*. and is not relevant to the choice of reflections for refinement. *R*-factors based on *F*^2^ are statistically about twice as large as those based on *F*, and *R*- factors based on all data will be even larger.

### Fractional atomic coordinates and isotropic or equivalent isotropic displacement parameters (Å^2^)

**Table:**

	*x*	*y*	*z*	*U*_iso_*/*U*_eq_	
O1	0.40817 (8)	0.80403 (18)	0.04185 (6)	0.0541 (3)	
N1	0.59419 (10)	0.8138 (2)	0.06169 (7)	0.0456 (3)	
H1N1	0.6036	0.9478	0.0317	0.055*	
C6	0.67241 (12)	0.6686 (2)	0.09809 (7)	0.0416 (3)	
N2	0.50984 (9)	0.51864 (19)	0.10975 (6)	0.0425 (3)	
C9	0.41188 (10)	0.3448 (2)	0.21353 (8)	0.0399 (3)	
C5	0.61861 (11)	0.4813 (2)	0.12841 (7)	0.0405 (3)	
C8	0.42295 (12)	0.3671 (2)	0.12812 (8)	0.0475 (4)	
H8A	0.4343	0.2127	0.1073	0.057*	
H8B	0.3565	0.4273	0.1029	0.057*	
C4	0.67330 (13)	0.3032 (3)	0.16849 (8)	0.0498 (4)	
H4	0.6375	0.1783	0.1886	0.060*	
C14	0.44588 (12)	0.5170 (2)	0.26587 (8)	0.0498 (4)	
H14	0.4778	0.6517	0.2487	0.060*	
C10	0.36386 (12)	0.1473 (3)	0.24098 (9)	0.0500 (4)	
H10	0.3403	0.0291	0.2069	0.060*	
C7	0.49498 (12)	0.7221 (2)	0.06784 (8)	0.0425 (3)	
C1	0.78212 (12)	0.6843 (3)	0.10756 (9)	0.0518 (4)	
H1	0.8181	0.8099	0.0879	0.062*	
C3	0.78367 (13)	0.3187 (3)	0.17738 (9)	0.0568 (4)	
H3	0.8228	0.2012	0.2039	0.068*	
C12	0.38486 (12)	0.2947 (3)	0.37129 (9)	0.0568 (4)	
C2	0.83703 (13)	0.5043 (3)	0.14782 (9)	0.0581 (4)	
H2	0.9113	0.5093	0.1550	0.070*	
C11	0.35059 (12)	0.1242 (3)	0.31856 (9)	0.0582 (4)	
H11	0.3178	−0.0095	0.3356	0.070*	
C13	0.43294 (13)	0.4911 (3)	0.34360 (9)	0.0580 (4)	
H13	0.4571	0.6084	0.3779	0.070*	
C15	0.37084 (17)	0.2654 (5)	0.45607 (11)	0.0944 (7)	
H15A	0.3991	0.4000	0.4839	0.142*	
H15B	0.4084	0.1281	0.4753	0.142*	
H15C	0.2962	0.2495	0.4628	0.142*	

### Atomic displacement parameters (Å^2^)

**Table:**

	*U*^11^	*U*^22^	*U*^33^	*U*^12^	*U*^13^	*U*^23^
O1	0.0523 (7)	0.0521 (6)	0.0563 (6)	0.0055 (5)	−0.0040 (5)	0.0144 (5)
N1	0.0529 (8)	0.0409 (6)	0.0424 (7)	0.0004 (5)	0.0018 (5)	0.0097 (5)
C6	0.0511 (9)	0.0410 (7)	0.0326 (7)	0.0025 (6)	0.0027 (6)	−0.0010 (5)
N2	0.0466 (7)	0.0404 (6)	0.0399 (6)	0.0001 (5)	0.0003 (5)	0.0076 (5)
C9	0.0376 (8)	0.0381 (7)	0.0431 (8)	0.0002 (5)	−0.0018 (6)	0.0029 (5)
C5	0.0484 (9)	0.0403 (7)	0.0323 (7)	0.0019 (6)	0.0006 (6)	−0.0010 (5)
C8	0.0535 (9)	0.0437 (7)	0.0440 (8)	−0.0072 (6)	−0.0024 (7)	0.0014 (6)
C4	0.0621 (10)	0.0427 (8)	0.0432 (8)	0.0042 (6)	−0.0029 (7)	0.0051 (6)
C14	0.0565 (10)	0.0425 (8)	0.0497 (9)	−0.0090 (6)	0.0009 (7)	−0.0003 (6)
C10	0.0496 (9)	0.0448 (8)	0.0542 (9)	−0.0094 (6)	−0.0019 (7)	0.0020 (6)
C7	0.0520 (9)	0.0398 (7)	0.0348 (7)	0.0029 (6)	0.0000 (6)	0.0033 (5)
C1	0.0519 (10)	0.0568 (9)	0.0471 (9)	−0.0046 (7)	0.0063 (7)	−0.0004 (6)
C3	0.0599 (11)	0.0558 (9)	0.0526 (9)	0.0145 (7)	−0.0063 (8)	0.0023 (7)
C12	0.0410 (9)	0.0808 (12)	0.0485 (9)	0.0004 (8)	0.0044 (7)	0.0068 (8)
C2	0.0490 (10)	0.0715 (11)	0.0529 (9)	0.0090 (8)	0.0000 (7)	−0.0049 (8)
C11	0.0481 (10)	0.0646 (10)	0.0619 (11)	−0.0107 (7)	0.0054 (8)	0.0173 (8)
C13	0.0599 (11)	0.0654 (10)	0.0478 (9)	−0.0044 (8)	−0.0003 (7)	−0.0104 (7)
C15	0.0829 (15)	0.148 (2)	0.0539 (12)	−0.0091 (14)	0.0142 (10)	0.0111 (12)

### Geometric parameters (Å, º)

**Table:**

O1—C7	1.2338 (16)	C14—C13	1.386 (2)
N1—C7	1.3649 (18)	C14—H14	0.9300
N1—C6	1.3933 (17)	C10—C11	1.383 (2)
N1—H1N1	0.9411	C10—H10	0.9300
C6—C1	1.375 (2)	C1—C2	1.392 (2)
C6—C5	1.3959 (19)	C1—H1	0.9300
N2—C7	1.3770 (16)	C3—C2	1.380 (2)
N2—C5	1.3913 (17)	C3—H3	0.9300
N2—C8	1.4521 (17)	C12—C11	1.381 (2)
C9—C14	1.3822 (19)	C12—C13	1.383 (2)
C9—C10	1.3857 (19)	C12—C15	1.512 (2)
C9—C8	1.5120 (19)	C2—H2	0.9300
C5—C4	1.3811 (18)	C11—H11	0.9300
C8—H8A	0.9700	C13—H13	0.9300
C8—H8B	0.9700	C15—H15A	0.9600
C4—C3	1.383 (2)	C15—H15B	0.9600
C4—H4	0.9300	C15—H15C	0.9600
			
C7—N1—C6	110.24 (11)	C9—C10—H10	119.7
C7—N1—H1N1	121.3	O1—C7—N1	127.42 (13)
C6—N1—H1N1	128.2	O1—C7—N2	125.97 (13)
C1—C6—N1	132.24 (13)	N1—C7—N2	106.61 (12)
C1—C6—C5	121.27 (13)	C6—C1—C2	117.16 (14)
N1—C6—C5	106.49 (12)	C6—C1—H1	121.4
C7—N2—C5	109.63 (11)	C2—C1—H1	121.4
C7—N2—C8	123.58 (12)	C2—C3—C4	121.57 (14)
C5—N2—C8	126.76 (11)	C2—C3—H3	119.2
C14—C9—C10	118.08 (13)	C4—C3—H3	119.2
C14—C9—C8	122.55 (12)	C11—C12—C13	117.44 (15)
C10—C9—C8	119.36 (12)	C11—C12—C15	120.94 (18)
C4—C5—N2	131.55 (13)	C13—C12—C15	121.61 (17)
C4—C5—C6	121.43 (14)	C3—C2—C1	121.44 (15)
N2—C5—C6	107.02 (11)	C3—C2—H2	119.3
N2—C8—C9	113.95 (11)	C1—C2—H2	119.3
N2—C8—H8A	108.8	C12—C11—C10	121.61 (15)
C9—C8—H8A	108.8	C12—C11—H11	119.2
N2—C8—H8B	108.8	C10—C11—H11	119.2
C9—C8—H8B	108.8	C12—C13—C14	121.43 (15)
H8A—C8—H8B	107.7	C12—C13—H13	119.3
C5—C4—C3	117.12 (14)	C14—C13—H13	119.3
C5—C4—H4	121.4	C12—C15—H15A	109.5
C3—C4—H4	121.4	C12—C15—H15B	109.5
C9—C14—C13	120.74 (14)	H15A—C15—H15B	109.5
C9—C14—H14	119.6	C12—C15—H15C	109.5
C13—C14—H14	119.6	H15A—C15—H15C	109.5
C11—C10—C9	120.69 (14)	H15B—C15—H15C	109.5
C11—C10—H10	119.7		
			
C7—N1—C6—C1	−179.97 (14)	C8—C9—C10—C11	−178.65 (14)
C7—N1—C6—C5	−0.56 (15)	C6—N1—C7—O1	−179.46 (13)
C7—N2—C5—C4	−179.25 (14)	C6—N1—C7—N2	1.05 (15)
C8—N2—C5—C4	−1.2 (2)	C5—N2—C7—O1	179.35 (13)
C7—N2—C5—C6	0.82 (14)	C8—N2—C7—O1	1.3 (2)
C8—N2—C5—C6	178.83 (12)	C5—N2—C7—N1	−1.15 (15)
C1—C6—C5—C4	−0.6 (2)	C8—N2—C7—N1	−179.24 (12)
N1—C6—C5—C4	179.90 (12)	N1—C6—C1—C2	−179.93 (14)
C1—C6—C5—N2	179.33 (12)	C5—C6—C1—C2	0.7 (2)
N1—C6—C5—N2	−0.16 (14)	C5—C4—C3—C2	0.3 (2)
C7—N2—C8—C9	−117.18 (14)	C4—C3—C2—C1	−0.1 (2)
C5—N2—C8—C9	65.08 (17)	C6—C1—C2—C3	−0.4 (2)
C14—C9—C8—N2	26.23 (19)	C13—C12—C11—C10	0.2 (2)
C10—C9—C8—N2	−155.20 (13)	C15—C12—C11—C10	−179.31 (17)
N2—C5—C4—C3	−179.82 (14)	C9—C10—C11—C12	−0.4 (2)
C6—C5—C4—C3	0.1 (2)	C11—C12—C13—C14	0.3 (2)
C10—C9—C14—C13	0.5 (2)	C15—C12—C13—C14	179.84 (16)
C8—C9—C14—C13	179.13 (14)	C9—C14—C13—C12	−0.7 (2)
C14—C9—C10—C11	0.0 (2)		

### Hydrogen-bond geometry (Å, º)

**Table:**

*D*—H···*A*	*D*—H	H···*A*	*D*···*A*	*D*—H···*A*
N1—H1*N*1···O1^i^	0.94	1.91	2.8317 (15)	166
C15—H15*C*···O1^ii^	0.96	2.58	3.514 (2)	165
C8—H8*A*···O1^iii^	0.97	2.61	3.5504 (18)	164

Symmetry codes: (i) −*x*+1, −*y*+2, −*z*; (ii) −*x*+1/2, *y*−1/2, −*z*+1/2; (iii) *x*, *y*−1, *z*.

## References

[bb1] Belaziz, D., Kandri Rodi, Y., Ouazzani Chahdi, F., Essassi, E. M., Saadi, M. & El Ammari, L. (2012). *Acta Cryst.* E**68**, o3212. 10.1107/S1600536812043620 PMC3515299 23284519

[bb2] Bruker (2009). *APEX2* and *SAINT* Bruker AXS Inc., Madison, Wisconsin, USA.

[bb3] Deligeorgiev, T., Kaloyanova, S. & Vasilev, S. (2011). *Dyes Pigm.* **90**, 170–1276.

[bb4] Farrugia, L. J. (2012). *J. Appl. Cryst.* **45**, 849–854.

[bb5] Gothelf, K. V. & Jørgensen, K. A. (1998). *Chem. Rev.* **98**, 863–909. 10.1021/cr970324e 11848917

[bb6] Lee, Y. H. & Pavlostathis, S. G. (2004). *Water Res.* **38**, 1838–1852. 10.1016/j.watres.2003.12.028 15026239

[bb7] Ouzidan, Y., Essassi, E. M., Luis, S. V., Bolte, M. & El Ammari, L. (2011). *Acta Cryst.* E**67**, o1822. 10.1107/S160053681102455X PMC3151763 21837191

[bb8] Scott, L. J., Dunn, C. J., Mallarkey, G. & Sharpe, M. (2002). *Drugs*, **62**, 1503–1538. 10.2165/00003495-200262100-00006 12093317

[bb9] Sheldrick, G. M. (2008). *Acta Cryst.* A**64**, 112–122. 10.1107/S0108767307043930 18156677

[bb10] Spek, A. L. (2009). *Acta Cryst.* D**65**, 148–155. 10.1107/S090744490804362X PMC2631630 19171970

[bb11] Westrip, S. P. (2010). *J. Appl. Cryst.* **43**, 920–925.

